# Angiopoietin‐2 Promotes Mechanical Stress‐induced Extracellular Matrix Degradation in Annulus Fibrosus *Via* the HIF‐1α/NF‐κB Signaling Pathway

**DOI:** 10.1111/os.13797

**Published:** 2023-07-21

**Authors:** Xiang Ao, Yuan Li, Tao Jiang, Chenglong Li, Zhengnan Lian, Liang Wang, Zhongmin Zhang, Minjun Huang

**Affiliations:** ^1^ Division of Spine Surgery, Department of Orthopaedics Nanfang Hospital of Southern Medical University Guangzhou Guangdong China; ^2^ Department of Spine Surgery, Center for Orthopedic Surgery The Third Affiliated Hospital of Southern Medical University Guangzhou Guangdong China; ^3^ Academy of Orthopaedics·Guangdong Province Guangzhou Guangdong China

**Keywords:** Angiopoietin‐2, Annulus fibrosus, HIF‐1α/NF‐κB signaling pathway, Intervertebral disc degeneration, Mechanical stress

## Abstract

**Objective:**

Mechanical stress is an important risk factor for intervertebral disc degeneration (IVDD). Angiopoietin‐2 (ANG‐2) is regulated by mechanical stress and is widely involved in the regulation of extracellular matrix metabolism. In addition, the signaling cascade between HIF‐1α and NF‐κB is critical in matrix degradation. This study aims to investigate the role and molecular mechanism of ANG‐2 in regulating the degeneration of annulus fibrosus (AF) through the HIF‐1α/NF‐κB signaling pathway.

**Methods:**

The bipedal standing mice IVDD model was constructed, and histological experiments were used to evaluate the degree of IVDD and the expression of ANG‐2 in the AF. Mouse primary AF cells were extracted *in vitro* and subjected to mechanical stretching experiments. Western blot assay was used to detect the effect of mechanical stress on ANG‐2, and the role of the ANG‐2‐mediated HIF‐1α/NF‐κB pathway in matrix degradation. In addition, the effect of inhibiting ANG‐2 expression by siRNA or monoclonal antibody on delaying IVDD was investigated at *in vitro* and *in vivo* levels. One‐way ANOVA with the least significant difference method was used for pairwise comparison of the groups with homogeneous variance, and Dunnett's method was used to compare the groups with heterogeneous variance.

**Results:**

In IVDD, the expressions of catabolic biomarkers (mmp‐13, ADAMTS‐4) and ANG‐2 were significantly increased in AF. In addition, p65 expression was increased while HIF‐1α expression was significantly decreased. The results of western blot assay showed mechanical stress significantly up‐regulated the expression of ANG‐2 in AF cells, and promoted matrix degradation by regulating the activity of HIF‐1α/NF‐κB pathway. Exogenous addition of Bay117082 and CoCl_2_ inhibited matrix degradation caused by mechanical stress. Moreover, injection of neutralizing antibody or treatment with siRNA to inhibit the expression of ANG‐2 improved the matrix metabolism of AF and inhibited IVDD progression by regulating the HIF‐1α/NF‐κB signaling pathway.

**Conclusion:**

In IVDD, mechanical stress could regulate the HIF‐1α/NF‐κB signaling pathway and matrix degradation by mediating ANG‐2 expression in AF degeneration.

## Graphical abstract

We discuss the expression of angiopoietin‐2 in annulus fibrosus during intervertebral disc degeneration, and the role of angiopoietin‐2 in regulating matrix degradation through the HIF‐1α/NF‐κB signaling pathway.

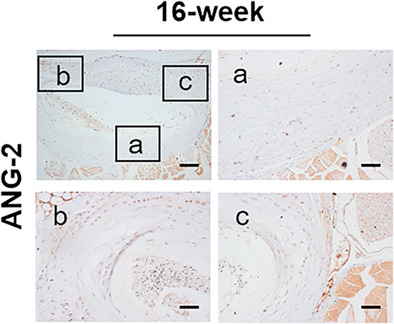

## Introduction

Intervertebral disc degeneration (IVDD) is a significant risk factor for spinal degenerative diseases, including low back pain and disc herniation.^1^, ^2^ Intervertebral disc (IVD) comprises a gel‐like nucleus pulposus (NP) and circumferentially annulus fibrosus (AF). Despite the high prevalence of AF injury in patients with low back pain and disability, few studies have focused on its injury and degeneration compared to the NP.^3^, ^4^ Excessive mechanical stress is a key factor in promoting IVDD.^5^ Epidemiological studies have demonstrated that individuals engaged in heavy physical labor have a significantly higher incidence of IVDD compared to the general population.^6^, ^7^ AF is the main structure that resists stress in IVD. Research using finite element analysis has identified the posterolateral part of the AF as a stress concentration area in the IVD, this area experiences significant changes in fiber strength and collagen composition, which are positively correlated with the degree of IVDD.^8^ Excessive stress has been found to cause an imbalanced metabolism of the extracellular matrix (ECM) in the AF.^9^ However, the molecular mechanism behind how mechanical stress regulates the ECM of the AF is yet to be fully understood.

Angiopoietin (ANG)‐2 is an important angiogenic factor that regulates vascular endothelial homeostasis.^10^ ANG‐2 competes with ANG‐1 to bind to the receptor Tie2, inhibits Tie2 phosphorylation and participates in the regulation of downstream signaling pathways, such as the PI3K/AKT and NF‐κB pathways.^11^, ^12^, ^13^ Studies in recent years have shown that ANG‐2 is involved in the progression of diseases in the musculoskeletal system.^14^, ^15^ Krausz *et al*. demonstrated that ANG‐2 promotes arthritis by promoting the inflammatory activation of macrophages.^15^ In addition, Wang *et al*. reported that ANG‐2 was expressed in the NP cells and promoted matrix degradation.^16^ However, the role and mechanism of ANG‐2 in the AF degeneration remain unclear. Previous studies have shown that the expression and function of ANG‐2 are closely related to mechanical stress in the vascular endothelial cells or the skeletal muscle.^17^, ^18^ Therefore, ANG‐2 may be an important target for mechanical stress‐induced AF degeneration.

The function of ANG‐2 is different under oxygenated and hypoxic conditions.^19^ Hypoxia‐inducible factor‐1 α (HIF‐1α) is an important transcription factor that regulates cell adaptation to hypoxia, suggesting that the function of ANG‐2 is closely related to HIF‐1α. The NF‐κB signaling pathway is an important downstream pathway of HIF‐1α,^20^, ^21^ and the HIF‐1α/NF‐κB signaling axis is involved in the regulation of multiple pathological processes such as colitis and spinal cord injury.^22^, ^23^ On the other hand, activation of the NF‐κB signaling pathway can lead to the matrix catabolism of IVD. Therefore, we hypothesized that excessive mechanical stress can mediate the HIF‐1α/NF‐κB signaling pathway by upregulating the expression of ANG‐2 in AF cells, thereby leading to disruption of the anabolic/catabolic homeostasis of ECM.^24^

In the previous study, we established a bipedal standing mouse IVDD model, which significantly promoted the progression of IVDD by increasing the axial loading of the IVD.^25^ In this study, the above mouse model was constructed and mouse primary AF cells were extracted to explore: (i) the effect of mechanical stress on the expression of ANG‐2; (ii) the effect of up‐regulated ANG‐2 on the metabolism of AF ECM; and (iii) The role of the HIF‐1α/NF‐κB signaling pathway in the above process.

## Materials and Methods

### Animal Experiments

Forty‐eight 12‐week‐old C57BL/6 male mice were purchased from the Experimental Animal Center of Southern Medical University. Mice were divided into the following groups according to the experimental design.

Experiment 1: in order to investigate the expression of ANG‐2 in the process of IVDD, 24 mice were randomly divided into the Ctrl group (n = 8) and the bipedal standing model group. The modeling method is briefly described as follows: the mice were placed in a separate modeling device, and then the modeling device was placed in a large water‐containing cage with a water depth of 5 mm, the model mice will actively maintain a bipedal standing posture. These mice stand 7 days a week for a total of 6 hours each day, the rest of time they took free activity as well as food and water intake.^24^ The bipedal standing mice were sacrificed at 12 weeks (12‐week group, n = 8) and 16 weeks after modeling (16‐week group, n = 8), respectively.

Experiment 2: in order to explore the protective effect of inhibiting ANG‐2 on the intervertebral disc, 24 mice were randomly divided into Ctrl group (n = 8) and bipedal standing model group. The modeling mice were intraperitoneally injected with normal saline (Saline group, n = 8) or ANG‐2 neutralizing antibody (1 mg/kg, ANG‐2 Nab group, n = 8) three times per week at the beginning of the modeling until 12 weeks, all mice were sacrificed 12 weeks after modeling to collect IVD specimens. The grouping information of the C57BL/6 mice is shown in Table 1.

**TABLE 1:** The grouping information of the C57BL/6 mice

	Group	Treatments	Number
Experiment 1	Ctrl group	None	8
	12‐week group	Bipedal standing modeling for 12 weeks	8
	16‐week group	Bipedal standing modeling for 16 weeks	8
Experiment 2	Ctrl group	None	8
	Saline group	Bipedal standing modeling for 12 weeks, injected with saline	8
	ANG‐2 NAb group	Bipedal standing modeling for 12 weeks, injected with ANG‐2 NAb	8

Another twenty 8‐week‐old C57BL/6 male mice were purchased for primary cell extraction. The animal experiments were approved by the Experimental Animal Ethics Committee of Southern Medical University (2019–1022), and the experimental mice were housed in a clean standard animal room at constant temperature and humidity.

### Imaging‐based Assessment

After mice were euthanized, lumbar spine samples (L3‐L6) were collected and fixed, followed by micro‐CT scanning (mCT 80, Scanco Medical, Bruttisellen, Zürich, Switzerland) using the following settings: 60 kV, 150 mA, and an average slice thickness of 20 mm. The disc height index (DHI%) of the three segments (L3/4, L4/5, and L5/6) was calculated and compared according to previous literature.^25^

### Histological Assessment

The IVD specimens were fixed, decalcified, dehydrated, and paraffin‐embedded to produce coronal and cross‐sectional continuous 4‐μm sections. After deparaffinization and hydration, hematoxylin and eosin (H&E) and Masson staining were performed to observe the degree of the IVDD. In coronal section, the cartilage endplate was divided into quarters according to the radial length, and the cartilage endplate heights at 25%, 50%, and 75% distances were measured and the average value was calculated as the cartilage endplate height of the sample. The IVDD histological score was calculated according to a modified scoring method.^26^

### Immunohistochemistry (IHC)

Sections were deparaffinized and hydrated and then placed in a citric acid solution to undergo 16 hours of incubation in a 60°C water bath for antigen retrieval, followed by incubation in a hydrogen peroxide solution for 10 minutes and blocking in 10% goat serum at 37°C for 30 minutes. The sections were then incubated in antibodies against p65, ADAMTS‐4 (purchased from ABclonal, Wuhan, China), Col1a2, MMP‐13, HIF‐1α (purchased from Abcam, Cambridge, MA, USA), and Col2a1 (purchased from Millipore, Burlington, MA, USA) at 4°C overnight, followed by incubation in secondary antibody (purchased from ABclonal, Wuhan, China) for 1 hour at room temperature, DAB chromogenesis, hematoxylin staining, and mounting to observe the expression of the corresponding proteins under a microscope.

### Primary Cell Culture

After the 8‐week‐old C57BL/6mice were sacrificed, the caudal vertebra tissues were collected and placed in high‐glucose Dulbecco's modified Eagle's medium (DMEM). The IVD tissue was separated and incised, the NP tissue was removed and the AF tissue was retained to transferred to another dish, and 0.2% type II collagenase was added for digestion at 37°C for 4 hours. The above operations were all completed under a stereomicroscope. After the digestion, the cell suspension was collected, centrifuged at 1000 rpm for 5 minutes, the supernatant was discarded, the cell pellet was washed three times with high‐glucose DMED medium, filtered through a 70 μm tissue filter, then DMEM/F12 medium supplemented with 10% fetal bovine serum (FBS; purchased from Gibco, Carlsbad, CA, USA) and 1% penicillin–streptomycin (purchased from Gibco) combination was used as culture medium at 37°C in a humidified atmosphere containing 5% CO_2_.

### RNA Interference

Using small interfering RNA (siRNA) technology combined with liposome co‐transfection method to knock down the expression of Angiopoietin2 protein in mouse AF cells. Three different siRNA oligonucleotide fragments (#1, #2, #3) were designed to ensure the efficiency. The cells were seeded in a 24‐well plate at a density of 0.5–2*10^5^. Lipofectamine™ 2000 (1 μL) and siRNA oligos (20 μm) were diluted with 50μL of Opti‐Minimal Essential Medium I (Cat no:31985062; Gibco) and incubated at room temperature for 5 min. After the incubation, the two were mixed gently and left at room temperature for 20 min to form the siRNA‐transfection reagent mixture. The protein expression level of ANG‐2 was detected by western blot 48 hours after transfection. After verifying the transfection effect, the AF cells seeded on the stretch plate were transfected for 48 hours, and the mechanical stretching experiment was performed.

### Application of Mechanical Stretch

The mouse AF cells were seeded in a 6‐well stretch plate, after the cell density reached 80%, the AF cells were subjected to mechanical stress stimulation using the FX5000 mechanical stretching system (Flexcell International Corp., Burlington, NC, USA. The schematic diagram of the equipment was shown in Supplementary Figure 1). Stretch for 0, 6, 12, and 24 hours with a 20% amplitude and a frequency of 0.1 Hz, respectively. Total cellular protein was subsequently extracted.

**Fig. 1:**
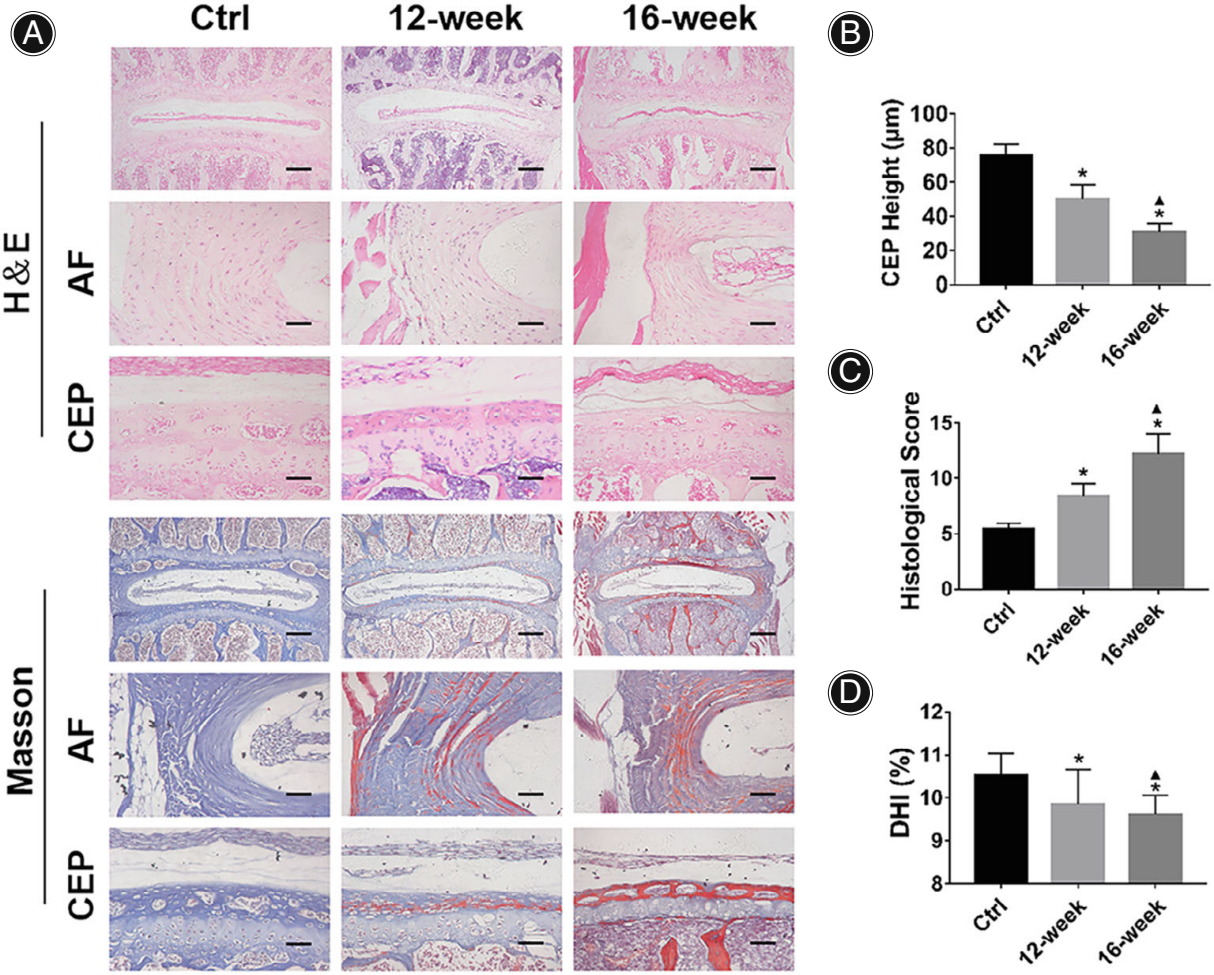
The degree of IVDD in the bipedal standing mouse model increased significantly. (A) The results of H&E staining showed that compared with the Ctrl group, the degree of IVDD increased in the mice of bipedal standing group with the prolongation of modeling time. The formation of AF fissures and the reduction of the cartilage endplate height were observed. The results of Masson staining showed that the red‐stained areas of the AF and cartilage endplate in the bipedal standing mice were increased with the prolongation of modeling time. (B) Statistical results of cartilage endplate height of mice in each group. (C) Statistical results of IVDD histological score of mice in each group. (D) Statistical results of DHI% of mice in each group. * indicates *P* < 0.05 compared with the Ctrl group, and ▲ indicates *P* < 0.05 compared with the 12‐week group, scale bar = 50 μm or 200 μm.

### Western Blot

Total protein was extracted and dissolved in Laemmli solution (62.5 mM Tris–HCl, pH 6.8, 2% sodium dodecyl sulfate, 10% glycerol, 50 mM dithiothreitol, and 0.01% bromophenol blue) for 10 min at 95°C. The proteins were separated by sodium dodecyl sulfate polyacrylamide gel electrophoresis and transferred to nitrocellulose membranes. The membrane strips were blocked at room temperature in 5% skim milk diluted with Tris‐buffered saline containing 0.1% Tween 20 for 1 h and incubated with antibodies against Col2a1, MMP‐13, ADAMTS‐4, HIF‐1α, β‐actin (purchased from Abcam, Cambridge, MA, USA), p65, IKB (purchased from Proteintech, Wuhan, China) overnight at 4°C. The strips were then incubated with a secondary antibody (purchased from ABclonal, Wuhan, China) at room temperature for 1 h. An ultrasensitive electrochemiluminescence kit was used to detect the bands, and software was used to image and analyze the bands.

### Data Analyses

SPSS 20.0 was used for statistical analysis. The data are expressed as the mean ± standard deviation. One‐way ANOVA with the least significant difference method was used for pairwise comparison of the groups with homogeneous variance, and Dunnett's method was used to compare the groups with heterogeneous variance. *P* < 0.05 indicates a statistically significant difference.

## Results

### The Degree of IVDD in the Bipedal Standing Mouse Model Increased Significantly

The results of H&E staining (Fig. 1A) showed that compared with the Ctrl group, the degree of IVDD in the mice of the12‐week and 16‐week groups was significantly increased, mainly manifested as: cell band compression in NP, fissures formation and increased hypertrophic chondrocytes in AF and decreased cartilage endplate height (Fig. 1B, Ctrl group: 75.66 ± 6.62 μm; 12‐week group: 50.16 ± 8.32 μm, *P* < 0.001; 16‐week group: 34.17 ± 5.45 μm, *P* < 0.001).

The results of histological score based on H&E staining (Fig. 1C) showed that compared with the Ctrl group (5.40 ± 0.55), the IVDD scores in the mice of the 12‐week group (8.40 ± 1.14, p = 0.003) and the 16‐week group (12.20 ± 1.79, p < 0.001) were significantly higher. The results of Masson staining showed that compared with the Ctrl group, the red‐stained areas in the AF and cartilage endplates of the 12‐week mice and 16‐week group mice were significantly increased. The bipedal standing group mice have lower intervertebral disc height according to the micro‐CT assessment (The CT images were shown in Supplementary Figure 2).The results of DHI% showed that compared with the Ctrl group (Fig. 1D, 10.53% ± 0.50%), the DHI% in the mice of 12‐week group (9.86% ± 0.80%, p = 0.038) and the 16‐week group decreased significantly (9.68% ± 0.25%, p = 0.016). The above results suggest that the degree of IVDD in mice of the bipedal standing group is significantly increased.

**Fig. 2:**
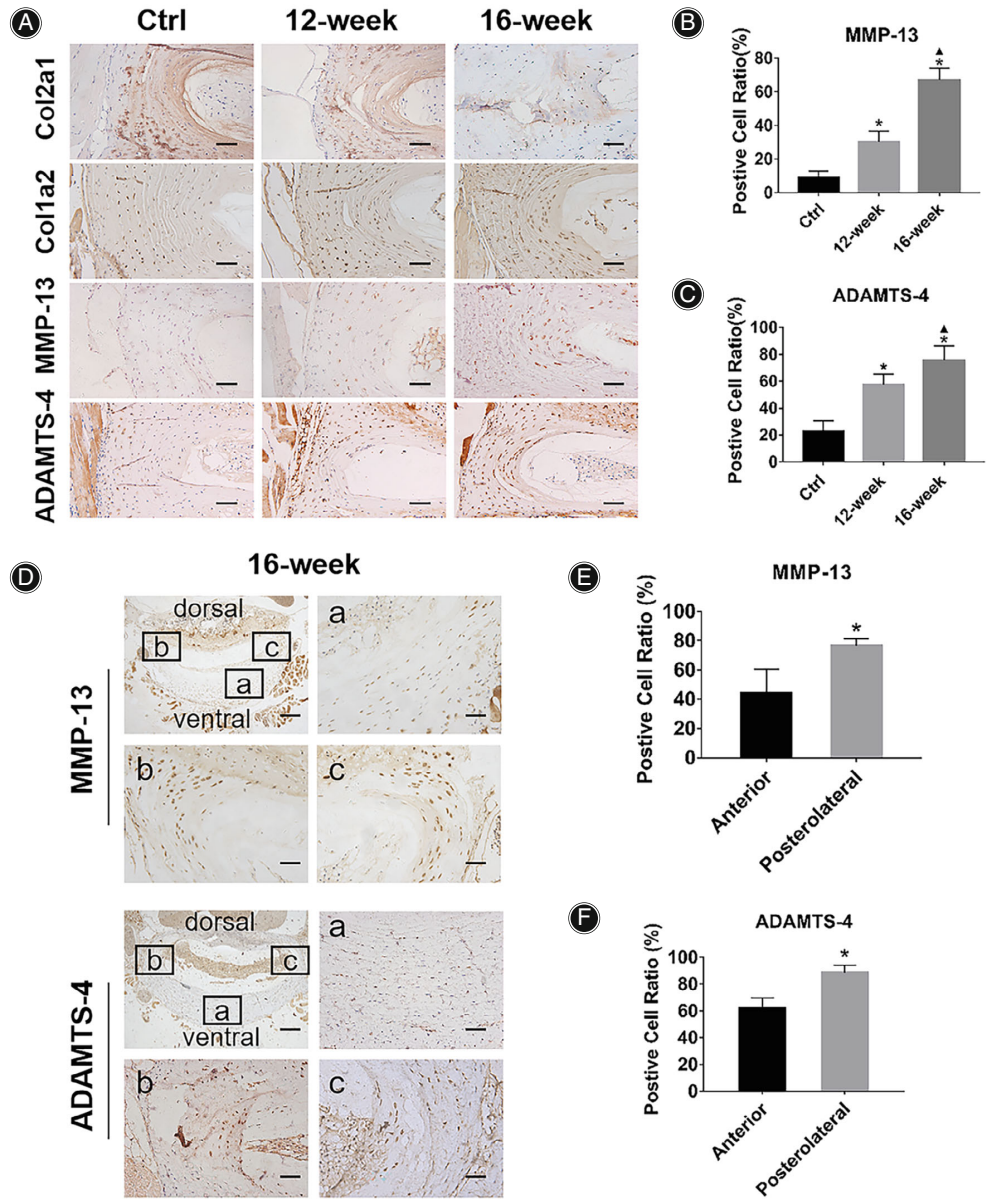
The ECM metabolic homeostasis of AF disrupted in the bipedal standing mice. (A) IHC results showed that compared with the Ctrl group, decreased expression of Col2a1 and increased expressions of Col1a2, MMP‐13 and ADAMTS‐4 in the AF of the bipedal standing mice were observed with the prolongation of modeling time. (B and C) Statistical results of positive cell ratio of MMP‐13 and ADAMTS‐4 in the AF of mice in each group. (D) IHC results showed that compared with the anterior AF, the expressions of MMP‐13 and ADAMTS‐4 were significantly increased in the posterolateral AF in the 16‐week group mice. (E and F) Statistical results of positive cell ratio of MMP‐13 and ADAMTS‐4 in the anterior region or posterolateral region of AF in 16‐week group mice. *Indicates *P* < 0.05 compared with the Ctrl group, and ▲indicates *p* < 0.05 compared with the 12‐week group, scale bar = 50 μm or 200 μm.

### Matrix Degradation of AF is Accelerated in the Bipedal Standing Mouse Model

IHC results (Fig. 2A) showed that compared with the Ctrl group, the expression of Col2a1 decreased and the expression of Col1 increased in the AF of the 12‐week and 16‐week group mice. MMP‐13 and ADAMTS‐4 are important matrix‐degrading enzymes. The IHC results showed that compared with the Ctrl group (MMP‐13: 9.17% ± 3.76%; ADAMTS‐4: 22.83% ± 7.76%), the positive cell ratios in the AF of the mice in the 12‐week group (MMP‐13: 30.33% ± 6.35%, *P* = 0.003; ADAMTS‐4: 57.5% ± 7.91%, *P* = 0.005) and the 16‐week group (MMP‐13: 67.17% ± 6.85%, *P*<0.001; ADAMTS‐4: 75.8% ± 10.35%, *P* < 0.001) was significantly increased (Fig. 2B, C).

Next, the expression of MMP‐13 and ADAMTS‐4in different regions of the AF was detected by IHC in the L4/5 disc (Fig. 2D). The result of MMP‐13 showed that the positive cell ratio was significantly higher in the posterolateral AF than in the anterior AF (Fig. 2E, 76.80% ± 4.55% *vs*. 44.03% ± 16.71%, *P* = 0.031). The result of ADAMTS‐4 was similar to MMP‐13, with significantly higher ratio of ADAMTS‐4‐positive cells in posterolateral AF compared to anterior AF (Fig. 2F, 88.77% ± 5.39% *vs*. 62.23% ± 7.39%, *P* = 0.007).

### Mechanical Stress Regulates ECM Metabolism by Upregulating ANG‐2 Expression in AF

**Fig. 3:**
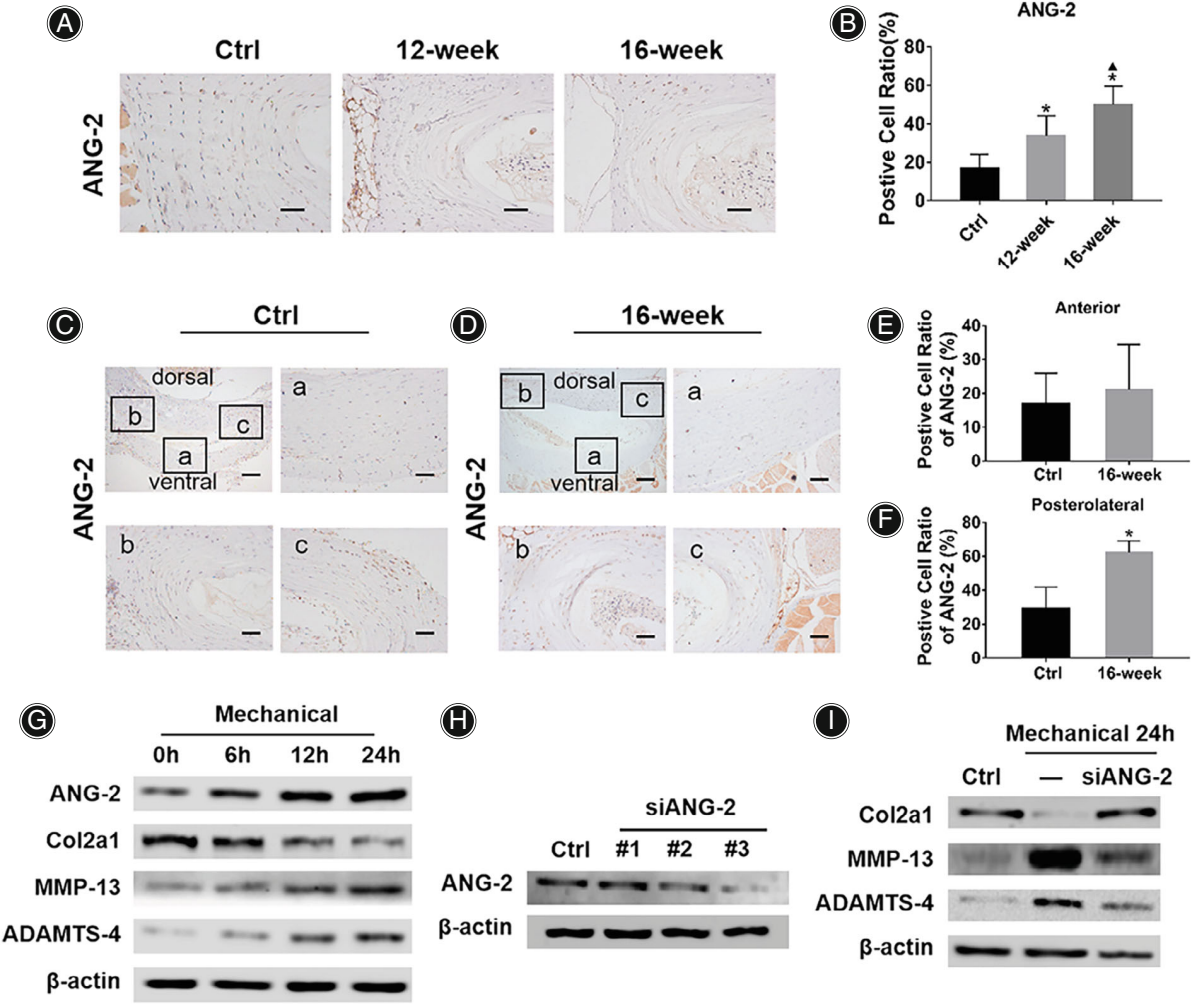
Mechanical stress upregulates ANG‐2 expression *in vivo* and *in vitro*. (A) IHC results showed that compared with the Ctrl group, the expression of ANG‐2 in the AF of mice in the 12‐week and 16‐week groups was increased. (B) Statistical results of the positive cell ratio of ANG‐2 in the AF of the mice in each group. (C and D) IHC results of ANG‐2 in the AF of the L4/5 IVD segment in the Ctrl group mice and the 16‐week group mice. (E) Statistical results of the positive cell ratio of ANG‐2 in the anterior AF region in the Ctrl group and 16‐week group mice. (F) Statistical results of the positive cell ratio of ANG‐2 in the posterolateral AF region in the Ctrl group and 16‐week group mice. (G) Western blot analysis of ANG‐2, Col2a1, MMP‐13, ADAMTS‐4 in AF cells treated with mechanical stretching. (H) Western blot analysis of ANG‐2 in AF cells treated with siANG‐2. (I) Western blot analysis of Col2a1, MMP‐13 and ADAMTS‐4 in AF cells treated with mechanical stretching or mechanical stretching plus siANG‐2. *Indicates *P* < 0.05 compared with the Ctrl group, and ▲indicates *P* < 0.05 compared with the 12‐week group, scale bar = 50 μm or 200 μm.

The IHC results showed that compared with the Ctrl group (16.95% ± 6.97%), the positive cell ratio of ANG‐2 in the AF of the mice in the 12‐week group (33.75% ± 10.37%, *P* = 0.029) and the 16‐week group was significantly increased (50.11% ± 9.59%, *P* = 0.001) (Fig. 3A, B). To further explore the relationship between mechanical stress and ANG‐2, we performed cross‐sectional slices of the L4/5 IVD, the IHC results showed that in the mice of Ctrl group and the 16‐week group, the expression of ANG‐2 was lower in the anterior AF region, while it was higher in the posterolateral AF region (Fig. 3C, D). Notably, compared with the Ctrl group (Fig. 3E, anterior: 16.92% ± 9.04%; posterolateral: 29.04% ± 12.91%), there was no significant difference in the positive cell ratio of ANG‐2 in the anterior AF region in the 16‐week group (21.06% ± 13.40%, *P* = 0.627), but the positive cell ratio in the posterolateral AF region was significantly increased (Fig. 3F, 62.43% ± 6.63%, *P*<0.001). The above results indicated that the expression of ANG‐2 was significantly increased in the stress concentration area of the AF.

Mechanical stretching was performed on the mouse AF cells, and the results of western blot assay showed that with the extension of the stretching time, the expressions of ANG‐2, MMP‐13 and ADAMTS‐4 gradually increased, while the expression of Col2a1 gradually decreased (Fig. 3G). siRNA transfection was performed to inhibit the expression of ANG‐2 in mouse AF cells. The results showed the inhibitory effect of sequence #3 was the most significant, so this sequence was selected for subsequent experiments (Fig. 3H). Further, the results of western blot assay showed that down‐regulated expression of ANG‐2 could significantly inhibit the catabolism of ECM induced by mechanical stress, which was manifested as increased expression of Col2a1 and decreased expression of MMP‐13 and ADAMTS‐4 (Fig. 3I).

### Mechanical Stress Upregulates NF‐κB Signaling Pathway Activity through ANG‐2

**Fig. 4:**
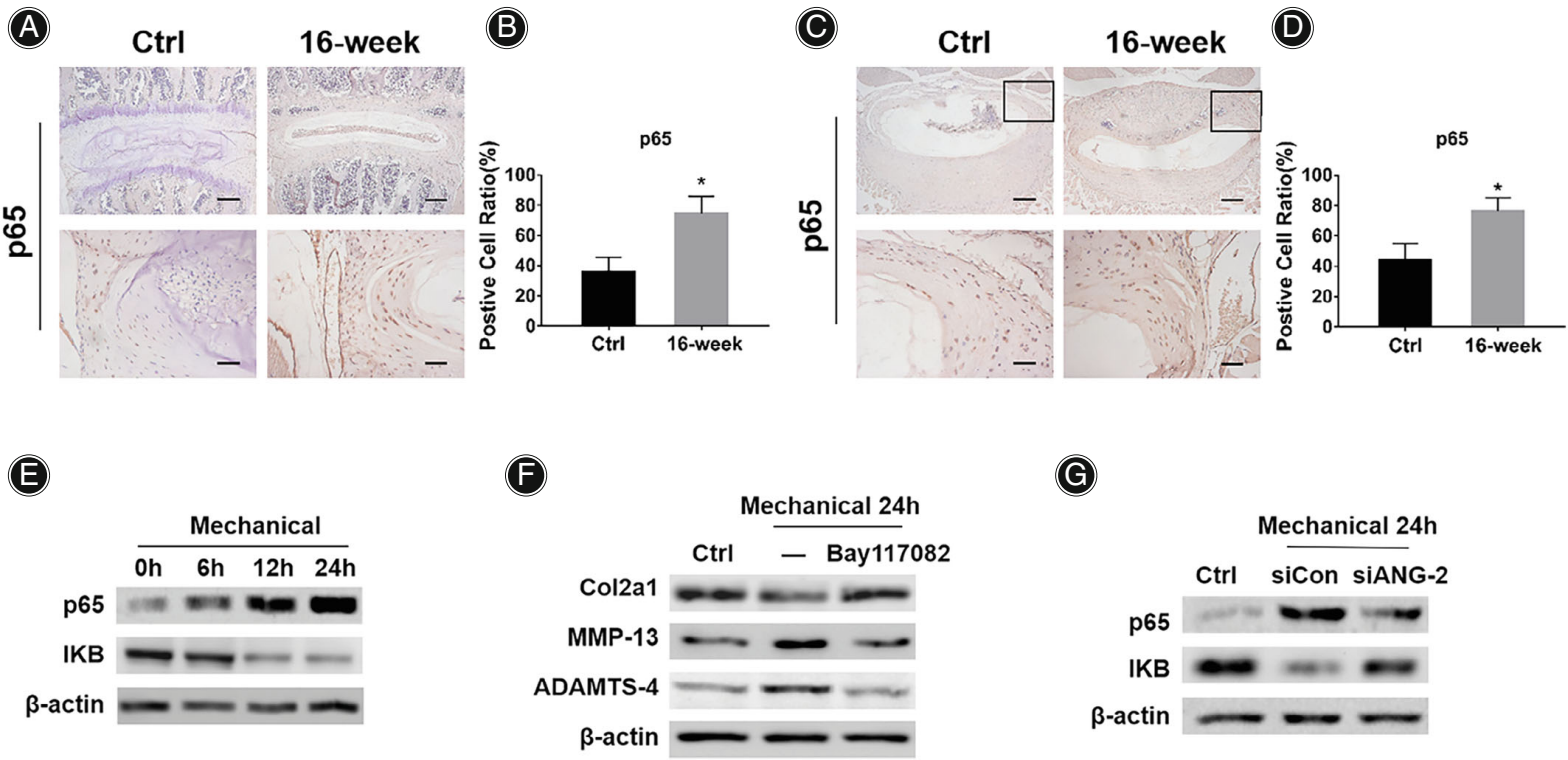
Mechanical stress modulates NF‐κB signaling pathway activity *in vivo* and *in vitro*. (A) IHC results of p65 in the AF of mice in the Ctrl group and the 16‐week group. (B) Statistical results of the positive cell ratio of p65 in the AF of mice in the Ctrl group and the 16‐week group. (C) IHC results of p65 in the posterolateral AF of the L4/5 IVD in the Ctrl group mice and the 16‐week group mice. (D) Statistical results of the positive cell ratio of p65 in the posterolateral AF of the L4/5 IVD in the Ctrl group mice and the 16‐week group mice. (E) Western blot analysis of p65 and IKB in AF cells treated with mechanical stretching. (F) Western blot analysis of Col2a1, MMP‐13 and ADAMTS‐4 in AF cells treated with mechanical stretching or mechanical stretching plus Bay117082. (G) Western blot analysis of p65 and IKB in AF cells treated with mechanical stretching or mechanical stretching plus siANG‐2. *Indicates *P* < 0.05 compared with the Ctrl group, scale bar = 50 μm or 200 μm.

The NF‐κB signaling pathway is an important molecular mechanism regulating ECM metabolism. IHC results showed that compared with the Ctrl group (Fig. 4A, B, 35.71% ± 10.02%), the positive cell ratio of p65 in the AF of the mice in the 16‐week group was significantly increased (74.04% ± 11.16%, p = 0.002). The IHC results of cross‐sectional L4/5 IVD showed that compared with the Ctrl group (Fig. 4C, D, 43.85% ± 11.07%), the positive cell ratio of p65 in the posterolateral AF region of the mice in the 16‐week group was significantly increased (76.51% ± 8.72%, p = 0.007).

Then mechanical stretching experiment was performed, the western blot results showed that the expression of p65 increased and the expression of IKB decreased in the mouse AF cells with the extension of the stretching time (Fig. 4E). The NF‐κB signaling pathway inhibitor Bay117082 was added to the stretched cells. The western blot results showed that Bay117082 could significantly inhibit the catabolism of ECM induced by mechanical stress, manifested as increased Col2a1 expression and decreased MMP‐13 and ADAMTS‐4 expressions (Fig. 4F). In further research, we used siRNA to inhibit the expression of ANG‐2 to explore the relationship between ANG‐2 and the NF‐κB signaling pathway. The western blot results showed that the down‐regulated ANG‐2 significantly inhibited the activity of the NF‐κB signaling pathway in mouse AF cells, manifested as a decrease in the expression of p65 and an increase in the expression of IKB (Fig. 4G).

### ANG‐2 Regulates NF‐κB Pathway through Mediating Expression of HIF‐1α

**Fig. 5:**
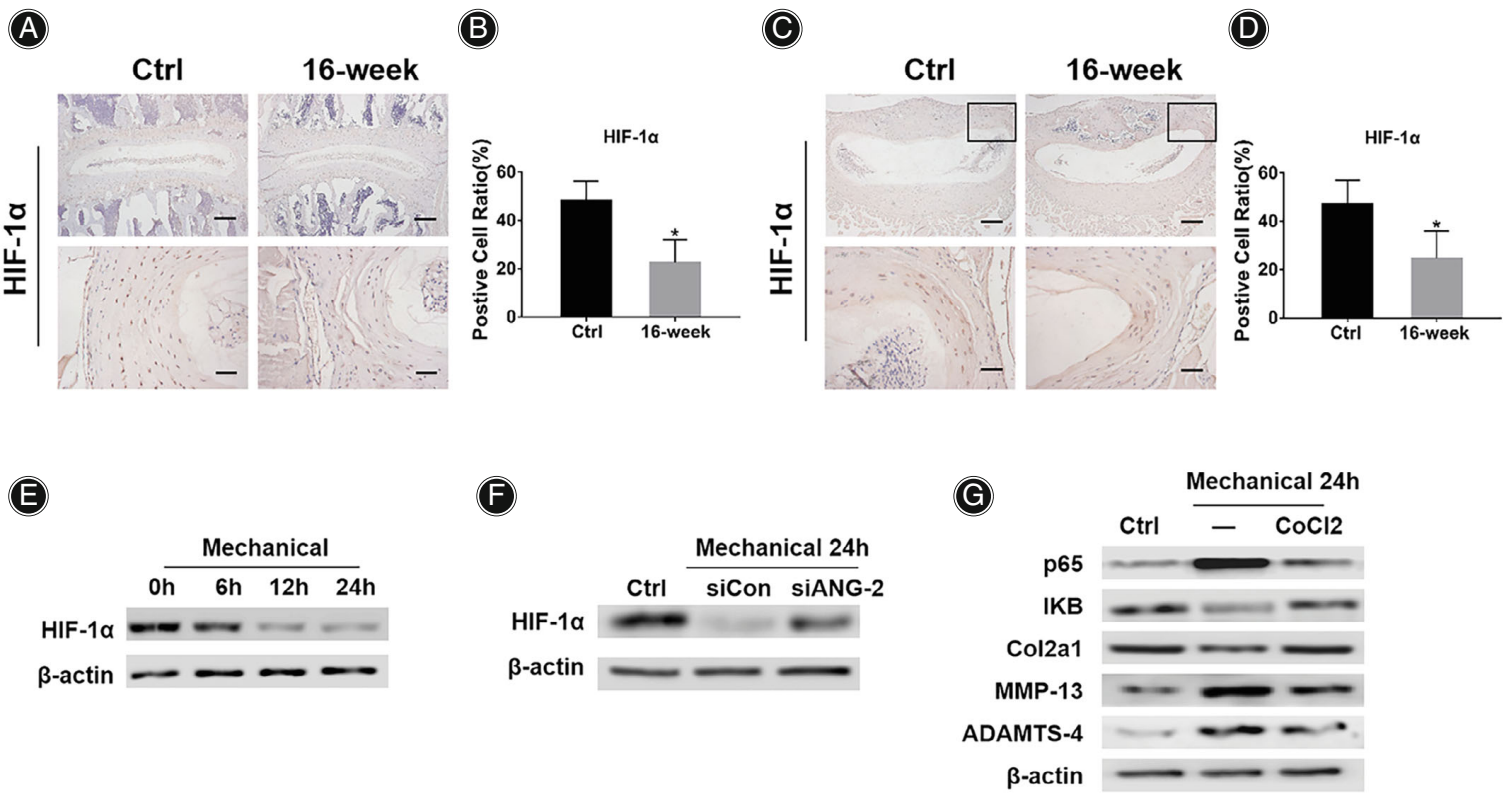
ANG‐2 regulates NF‐κB pathway through mediating expression of HIF‐1α in AF. (A) IHC results of HIF‐1α in the AF of mice in the Ctrl group and the 16‐week group. (B) Statistical results of the positive cell ratio of HIF‐1α in the AF of mice in the Ctrl group and the 16‐week group. (C) IHC results of HIF‐1α in the posterolateral AF of the L4/5 IVD in the Ctrl group mice and the 16‐week group mice. (D) Statistical results of the positive cell ratio of HIF‐1α in the posterolateral AF of the L4/5 IVD in the Ctrl group mice and the 16‐week group mice. (E) Western blot analysis of HIF‐1α in AF cells treated with mechanical stretching. (F) Western blot analysis of HIF‐1α in AF cells treated with mechanical stretching or mechanical stretching plus siANG‐2. (G) Western blot analysis of p65, IKB, Col2a1, MMP‐13 and ADAMTS‐4 in AF cells treated with mechanical stretching or mechanical stretching plus CoCl_2_. *Indicates *P* < 0.05 compared with the Ctrl group, scale bar = 50 μm or 200 μm.

Since HIF‐1α is implicated in inflammation inhibition, we explored the relationship between the HIF‐1α and NF‐κB signaling pathways in AF. IHC results showed that compared with the Ctrl group (Fig. 5A, B, 48.07% ± 8.19%), the positive cell ratio of HIF‐1α in the AF of the mice in the 16‐week group was significantly decreased (22.41% ± 9.56%, *P* = 0.001). The IHC results of cross‐sectional L4/5 IVD showed that compared with the Ctrl group (Fig. 5C, D, 47.38% ± 9.60%), the positive cell ratio of HIF‐1α in the posterolateral AF region of the mice in the 16‐week group was significantly decreased (24.62% ± 11.44%, p = 0.011).

Western blot results showed that the expression of HIF‐1α in mouse AF cells decreased with the extension of stretching time (Fig. 5E). However, the treatment of siANG‐2 effectively up‐regulated the expression of HIF‐1α in stretched AF cells, suggesting that the activity of HIF‐1α was regulated by the upstream ANG‐2 (Fig. 5F). CoCl_2_ can significantly increase the expression of HIF‐1α. In this study, addition of CoCl2 to stretched mouse AF cells effectively inhibited the activity of the NF‐κB signaling pathway, manifested as decreased p65 expression and increased IKB expression, suggesting HIF‐1α is an important target that mediates ANG‐2 regulation of the NF‐κB signaling pathway. Furthermore, the addition of CoCl2 effectively improved the anabolic metabolism of AF under mechanical stretching, which was manifested as increased Col2a1 expression and decreased MMP‐13 and ADAMTS‐4 expressions (Fig. 5G).

### Injection of ANG‐2 Neutralizing Antibody Effectively Inhibited IVDD in Vivo

**Fig. 6:**
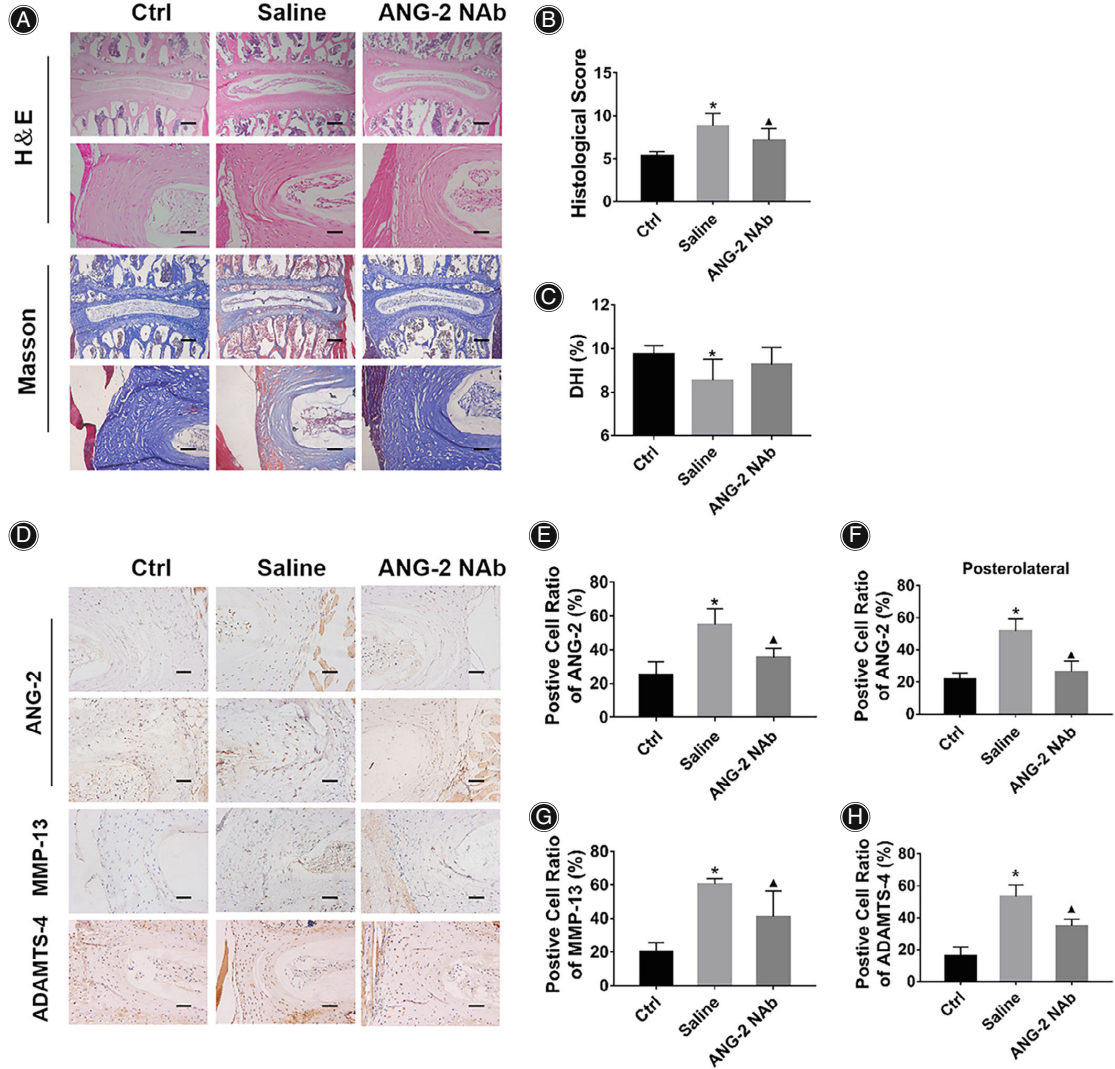
Injection of ANG‐2 neutralizing antibody inhibited IVDD. (A) Histological staining results showed that compared with the Ctrl group, the degree of IVDD in the mice of Saline group was significantly increased, while the injection of ANG‐2 neutralizing antibody inhibited the progression of IVDD, including reduced AF fissures, and reduced red‐stained areas in Masson staining. (B) Statistical results of IVDD histological score of mice in each group. (C) Statistical results of DHI% of mice in each group. (D) IHC results showed that compared with the Ctrl group, the expressions of ANG‐2, MMP‐13 and ADAMTS‐4 in the AF of Saline group mice were increased, and the injection of ANG‐2 neutralizing antibody inhibited the expression of the above proteins. (E) Statistical results of the positive cell ratio of ANG‐2 in AF of mice in each group. (F) Statistical results of positive cell ratio of ANG‐2 in the posterolateral AF of the L4/5 IVD in each group. (G and H) Statistical results of positive cell ratio of MMP‐13 and ADAMTS‐4 in the AF of mice in each group. *Indicates *P* < 0.05 compared with the Ctrl group, and ▲indicates *P* < 0.05 compared with the Saline group, scale bar = 50 μm or 200 μm.

In order to examine the effect of inhibiting ANG‐2 expression on IVDD *in vivo*, the bipedal standing mice were injected with saline or ANG‐2 neutralizing antibody (ANG‐2 Nab), respectively. The results of H&E staining showed that compared with the Saline group, the degree of IVDD in the mice of ANG‐2 Nab group was reduced, the fissures and hypertrophic chondrocytes in AF were decreased. The results of Masson staining showed that injection of ANG‐2 Nab reduced the red‐stained fibrous area in AF (Fig. 6A). The results of IVDD histological score showed that compared with the Ctrl group (Fig. 6B, 5.33 ± 0.51), the score of the mice in the Saline group was significantly higher (8.83 ± 1.47, *P*<0.001), while the score of the mice in the ANG‐2 NAb group was significantly lower than that of the mice in the Saline group (7.20 ± 1.33, *P* = 0.029). Micro‐CT results showed that compared with the Ctrl group (The CT images were shown in Supplementary Figure 3; Fig. 6C, 9.77% ± 0.37%), the DHI% of the mice in the Saline group was significantly lower (8.54% ± 0.99%, *P* = 0.012), while the injection of ANG‐2 NAb could effectively maintain the DHI% of the mice (9.27% ± 0.82%), and its DHI was significantly higher than that of the mice in the Saline group (*P* = 0.091).

The IHC results (Fig. 6D) showed that compared with the Ctrl group mice (20.06% ± 4.50%), the positive cell ratio of ANG‐2 in the AF of the Saline group mice was significantly increased (Fig. 6E, 51.68% ± 7.62%, *P*<0.001), and the injection of ANG‐2 NAb significantly reduced the positive cell ratio of ANG‐2 (26.60% ± 4.61, *P*<0.001). The IHC results of the cross‐sectional L4/5 IVD showed in the posterolateral AF region, the positive cell ratio of ANG‐2 in the Saline group mice was significantly higher than that in the Ctrl group mice (Fig. 6F, 54.80% ± 9.52% *vs* 25.38% ± 7.39%, *P*<0.001), and the injection of ANG‐2 NAb significantly reduced the positive cell ratio of ANG‐2 (35.49% ± 5.42%, *P* = 0.004). The results of MMP‐13 and ADAMTS‐4 in the AF showed that compared with the Ctrl group mice (Fig. 6G, MMP‐13: 27.97% ± 4.38%; Fig. 6H, ADAMTS‐4: 20.50% ± 7.41%), the positive cell ratios in the Saline group mice were significantly increased (MMP‐13: 42.80% ± 2.57%, *P*<0.001; ADAMTS‐4: 68.00% ± 6.83%, *P*<0.001), and the injection of ANG‐2 NAb significantly reduced the positive cell ratio of these biomarkers of IVDD (MMP‐13: 35.38% ± 8.59%, *P* = 0.001; ADAMTS‐4: 51.25% ± 5.97%, *P* = 0.004). The above results indicate that injection of ANG‐2 Nab effectively inhibited the IVDD progression and the catabolism of AF *in vivo*.

### Injection of ANG‐2 Neutralizing Antibody Reversed HIF‐1α/NF‐κB Signaling Pathway Activity in Vivo

**Fig. 7:**
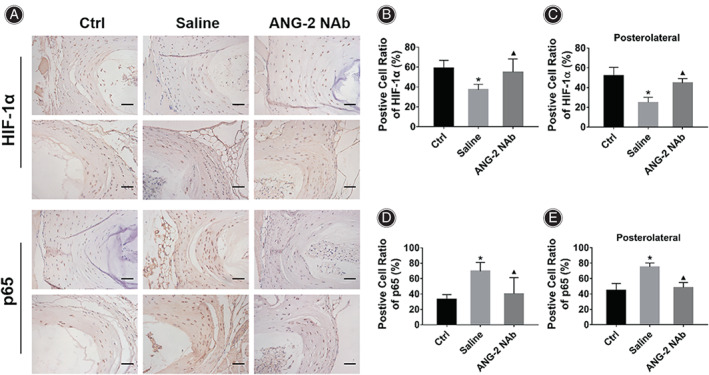
Injection of ANG‐2 neutralizing antibody reversed HIF‐1α/NF‐κB signaling pathway activity. (A) IHC results showed that compared with the Ctrl group, the expression of Hif‐1a was decreased, and the expression of p65 was increased in the AF of the mice in the Saline group. Injection of ANG‐2 neutralizing antibody upregulated the expression of HIF‐1α and inhibited the expression of p65 in the AF. (B) Statistical results of the positive cell ratio of HIF‐1α in the AF of mice in each group. (C) Statistical results of positive cell ratio of HIF‐1α in the posterolateral AF of the L4/5 IVD in each group. (D) Statistical results of the positive cell ratio of p65 in the AF of mice in each group. (E) Statistical results of positive cell ratio of p65 in the posterolateral AF of the L4/5 IVD in each group. *Indicates *P* < 0.05 compared with the Ctrl group, and ▲indicates *P* < 0.05 compared with the Saline group, scale bar = 50 μm or 200 μm.

IHC results (Fig. 7A) showed that compared with the Ctrl group mice (HIF‐1α: 58.75% ± 8.11%; p65: 33.21% ± 5.92%), the positive cell ratio of HIF‐1α in the AF of the Saline group mice was significantly decreased (Fig. 7B, 37.15% ± 5.58%, *P* = 0.011), and the positive cell ratio of p65 was significantly increased (Fig. 7D, 69.64% ± 11.43%, *P* = 0.005). The injection of ANG‐2 NAb significantly increased the positive cell ratio of HIF‐1α (54.98% ± 13.25%, *P* = 0.027) and reduced the positive cell ratio of p65 in the AF (40.49% ± 20.88%, *P* = 0.008). The differences were also significant in the posterolateral AF region of the L4/5 IVD, the results exhibited that compared with the Ctrl group mice (Fig. 7C, HIF‐1α: 51.86% ± 8.69%; Fig. 7E, p65: 44.85% ± 8.98%), the positive cell ratio of HIF‐1α was significantly lower (24.95% ± 5.21%, *P*<0.001) and the positive cell ratio of p65 was significantly higher (75.26% ± 4.86%, *P*<0.001) in the Saline group mice. The injection of ANG‐2 NAb significantly increased HIF‐1α (44.68% ± 4.54%, *P* = 0.004) and reduce p65 expression (48.24% ± 6.64%, *P*<0.001).

## Discussion

Excessive degradation of ECM in AF tissue is an important hallmark of IVDD. Previous research indicated ANG‐2 regulates the proliferation and adhesion of NP cells, then promotes its apoptosis and matrix degradation.^27^ This study aimed to explore the expression pattern and physiopathological role of ANG‐2 in AF degeneration. The results showed that mechanical stress significantly upregulated the expression of ANG‐2 in the AF cells and promote matrix breakdown through the HIF‐1α/NF‐κB signaling pathway. Neutralizing antibody injection or siRNA treatment can effectively inhibit the expression of ANG‐2 in the AF cells and improve the metabolic homeostasis of ECM.

### Effect of Mechanical Stress on the Expression of ANG‐2

Mechanical stress is an important risk factor for IVDD. Numerous studies have shown that excessive mechanical stress can inhibit cellular activity, promote matrix degradation, and accelerate IVDD.^28^, ^29^, ^30^ Previous study has shown that the expression of ANG‐2 is significantly increased in the degenerative NP tissue. However, the expression of ANG‐2 and its relationship to mechanical stress in the AF tissue during IVDD was still unclear. In this study, we established a bipedal standing mouse IVDD model. The IHC results showed that with the prolongation of modeling time, the degree of IVDD and the expression of ANG‐2 in AF were significantly increased. Hobohm *et al*. showed that the expression of ANG‐2 in vascular endothelial cells is regulated by mechanical stress.^10^ In addition, the finite element analysis has showed that the posterolateral AF region of IVD suffered significantly increased mechanical force in comparison with the other region, for example, the anterior.^31^ In order to explore the relationship between ANG‐2 and mechanical stress in AF cells, we performed the IHC experiment on the cross‐sectional slices of the L4/5 IVD. The results showed that compared with the Ctrl group, the expression of ANG‐2 in the anterior AF region of the mice in the 16‐week group had no significant difference. However, the expression of ANG‐2 was significantly increased in the posterolateral AF regions, indicating that mechanical stress is involved in regulating the expression of ANG‐2 in AF. *In vitro* experiments also confirmed that mechanical stress can up‐regulate the expression of ANG‐2 in mouse AF cells in a time‐dependent manner. The above results delineated the spatial specificity of ANG‐2 expression in the AF, which is closely related to the effects of mechanical stress. Previous studies have shown that many Mechan transduction mechanisms can regulate the expression of ANG‐2, including integrin, YAP and Ca^2+^ influx.^27^, ^32^, ^33^ However, the specific Mechan transduction mechanism regulating ANG‐2 in IVDD remains for further study.

### Effect of ANG‐2 on the Metabolism of ECM

ANG‐2 was highly expressed in the posterolateral AF of degenerated IVD, which is a common site of intervertebral disc herniation, suggesting that ANG‐2 plays an important role in regulating matrix degradation.^31^ Hou *et al*. reported that ANG‐2 can significantly upregulate MMP‐9 and MMP‐2 in trophoblast cells and promote matrix degradation.^34^ In this study, the results showed that in the AF of the bipedal standing mice, the expression of Col2a1was decreased, the expressions of MMP‐13 and ADAMTS‐4 were increased and showed similar spatial specificity to ANG‐2. It indicated that the disruption of matrix homeostasis in degenerated AF tissue was closely related to ANG‐2. The injection of ANG‐2 neutralizing antibodies can significantly inhibit the above pathological changes, and the treatment of siANG‐2 can also effectively improve the matrix metabolism of mouse AF cells. The above results demonstrated that ANG‐2 is an important link in the regulation of AF matrix metabolism by mechanical stress, suggesting that ANG‐2 can be used as a potential target for prevention and treatment of IVDD.

### The Role of HIF‐1α/NF‐κB Signaling Pathway in IVDD

The NF‐κB signaling pathway plays an important role in the regulation of IVD matrix metabolism.^24^ The results of this study show that mechanical stress can significantly upregulate the NF‐κB signaling pathway, which is consistent with previous studies. Wu *et al*. showed that mechanical stress upregulates NF‐κB signaling pathway and mediates NP cell senescence and catabolism by activating Piezo1.^35^ In this study, inhibition of ANG‐2 expression by injection of neutralizing antibodies or siRNA can down‐regulate the activity of the NF‐κB signaling pathway *in vivo* and *in vitro* and improve the ECM metabolism of AF, suggesting that ANG‐2 plays the key role in the NF‐κB signaling pathway. HIF‐1α is an important upstream signal of the NF‐κB signaling pathway, and HIF‐1α/NF‐κB is widely involved in the pathological process of oxidative stress and inflammatory injury.^15^, ^36^ Previous studies have shown that the function of ANG‐2 is closely related to the hypoxic environment, suggesting that HIF‐1α may be a key factor mediating ANG‐2 regulation of NF‐κB.^37^ Indeed, HIF‐1α is closely related to the NF‐κB signaling pathway. Yang *et al*. showed that Roxadustat inhibited the activity of the NF‐κB signaling pathway by up‐regulating HIF‐1α, this effect can be blocked by transfection siRNA of HIF‐1α, suggesting that HIF‐1α is the upstream factor of the NF‐κB signaling pathway.^20^ In addition, HIF‐1α depletion activated NF‐κB signaling in a CDK6‐dependent manner.^38^ In this study, the expression of HIF‐1α in the degenerated AF was significantly decreased. *In vitro* experiments showed the treatment of siANG‐2 blocked the inhibitory effect of mechanical stress on HIF‐1α. Meanwhile, using CoCl2 to upregulate the expression of HIF‐1α significantly inhibited NF‐κB signaling pathway activity. The above results indicate that the HIF‐1α/NF‐κB signaling pathway is an important regulator for ANG‐2 to mediate the ECM metabolism of AF, which further enriches the mechanism of mechanical stress‐induced IVDD.

### Limitation and Strengths

This study still has the following limitations, however. First, we only observed the expression changes of ANG‐2 in the IVD specimens of experimental mice, in follow‐up studies, clinical IVD specimens need to be included for deeper analysis. Second, mice were treated with ANG‐2 NAb by intraperitoneal injection in this study, in the future, it will be necessary to perform more precise targeted inhibition on the IVDD model of large animals. Nevertheless, this study explores the molecular mechanism and key targets of excessive degradation of the ECM in the AF, which can provide a theoretical basis for the clinical prevention and treatment of IVDD and low back pain.

## Conclusion

In summary, this study used the bipedal standing mouse model and mechanical stretch experiments of mouse AF cells, confirmed that mechanical stress up‐regulates the expression of ANG‐2 in AF cells, and disrupted its ECM metabolism by regulating the HIF‐1α/NF‐κB signaling pathway. Our study revealed a new mechanism by which mechanical stress promotes IVDD and AF matrix degradation. Moreover, ANG‐2 may serve as a potential target for the treatment of mechanical stress‐induced IVDD.

## Author contributions

Conceptualization, M.H. L.W. and Z.Z.; methodology, X.A. and Z.L.; software, Y.L.; validation, X.A., Z.L. and M.Z.; formal analysis, C.L.; investigation, X.A.; resources, Z.L.; data curation, Y.L.; writing—original draft preparation, X.A.; writing‐review and editing, M.H.; funding acquisition, M.H. and Z.Z. All authors have read and agreed to the published version of the manuscript.

## Funding Information

This work was funded by National Natural Sciences Foundation of China (82072520, 81874013[ZZ]), Natural Science Foundation of Guangdong Province (2019A1515012074 [MH]).

## Conflicts of interest

All authors declare no competing financial interests.

## Ethical statement

All animal experiments performed in this study were in accordance with the relevant guidelines of the Experimental Animal Ethics Committee of Southern Medical University.

## Supporting information


**Supplementary figure 1:** The schematic diagram of the Flexcell cell stretching system. A: The display system of the Flexcell cell stretching system; B: Placement table for cell stretching plate; C: Schematic diagram of cell stretch placement in a cell incubator; D: Operation interface diagram of flexcell system.

**Supplementary figure 2:** The CT images of the lumbar spine tissues of the mice in the Ctrl group, the 12‐week group and the 16‐week group.

**Supplementary figure 3:** The CT images of the lumbar spine tissues of the mice in the Ctrl group, the Saline group and the ANG‐2 NAb group.

## Data Availability

The data used to support the findings of this study are available from the corresponding author upon request.
