# Supplementary figures and images for: Angiopoietin‐2 Promotes Mechanical Stress‐induced Extracellular Matrix Degradation in Annulus Fibrosus Via the HIF‐1α/NF‐κB Signaling Pathway
Source: Orthop Surg. 2023 Jul 21;15(9):2410–22. doi: 10.1111/os.13797 (PMC10475680; doi:10.1111/os.13797)

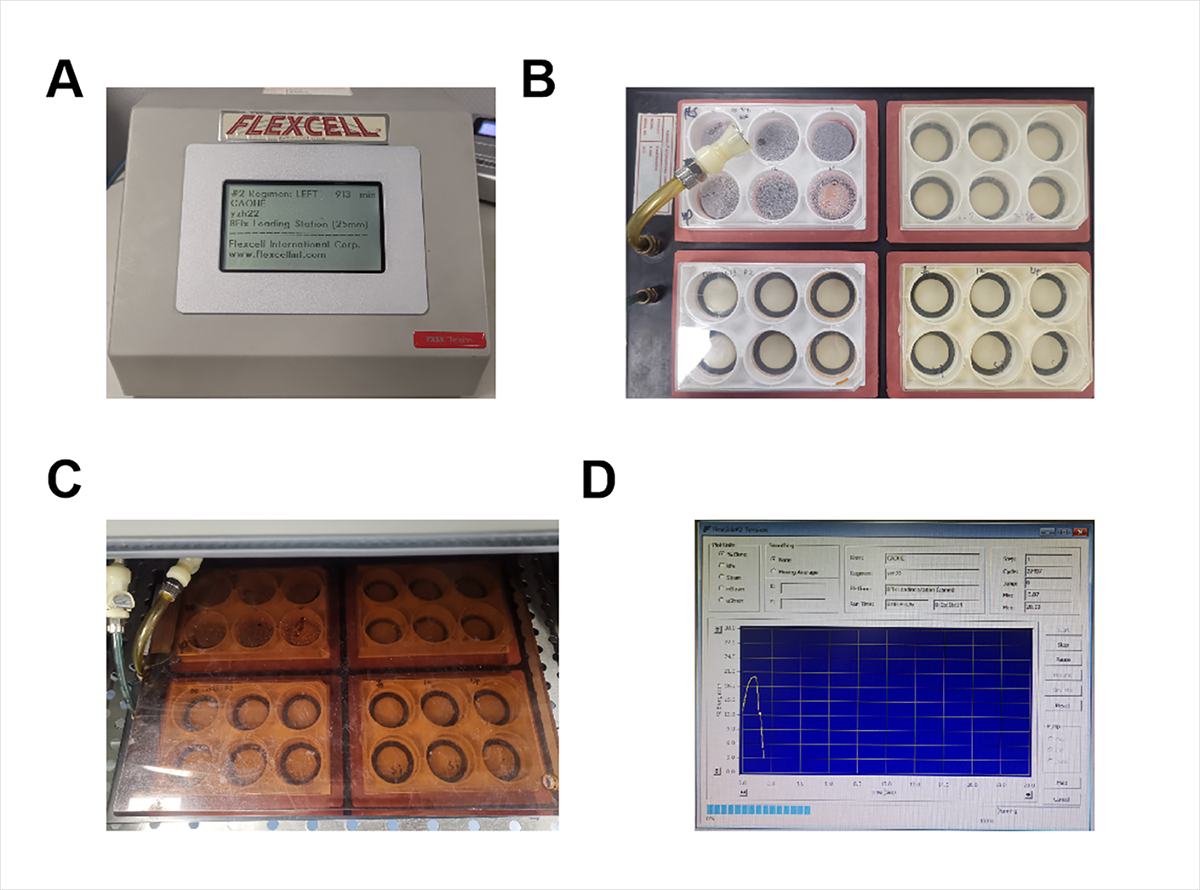

Supplement: Supplementary file 1 — Supplementary figure 1: The schematic diagram of the Flexcell cell stretching system. A: The display system of the Flexcell cell stretching system; B: Placement table for cell stretching plate; C: Schematic diagram of cell stretch placement in a cell incubator; D: Operation interface diagram of flexcell system. [file OS-15-2410-s003.tif]

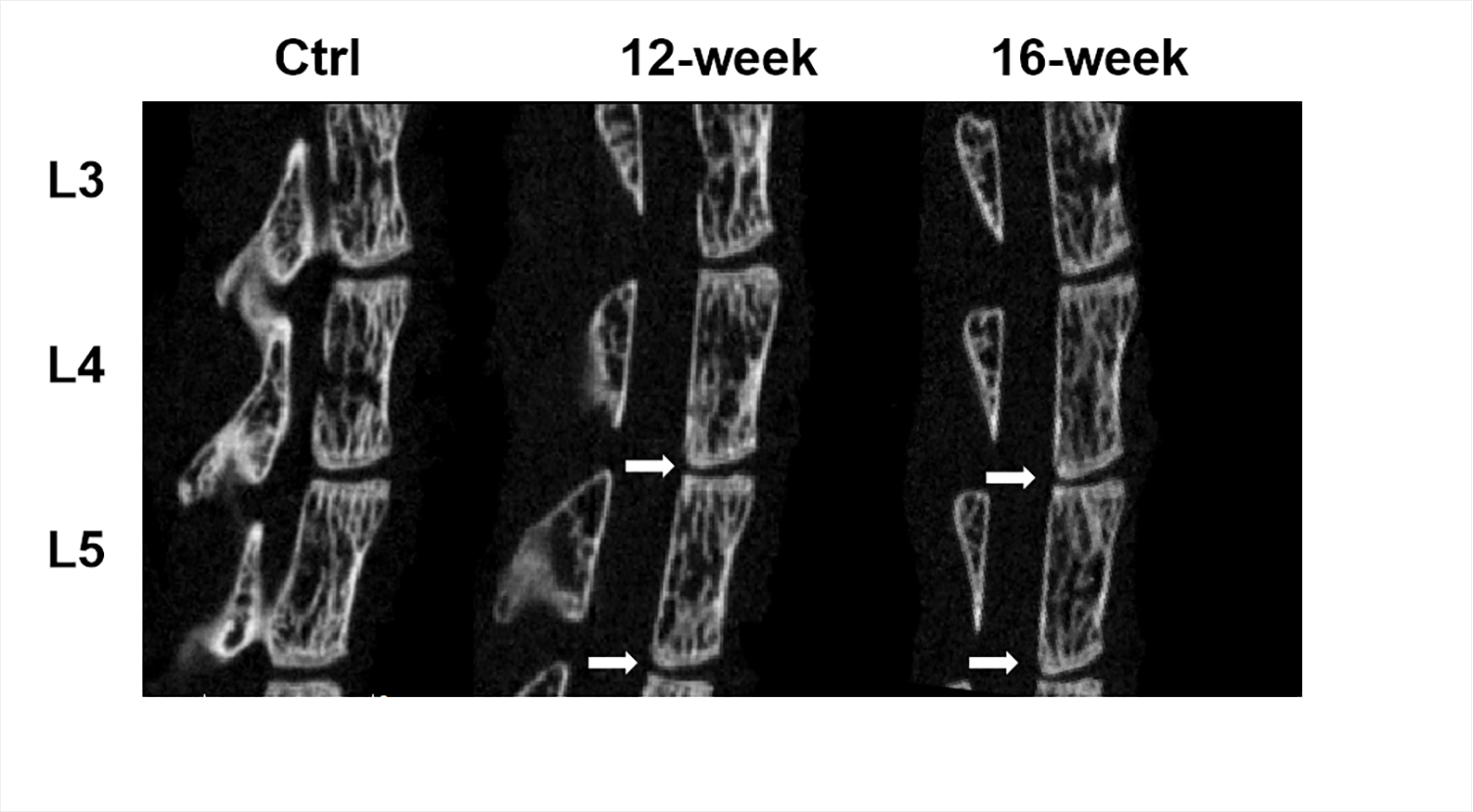

Supplement: Supplementary file 2 — Supplementary figure 2: The CT images of the lumbar spine tissues of the mice in the Ctrl group, the 12‐week group and the 16‐week group. [file OS-15-2410-s002.tif]

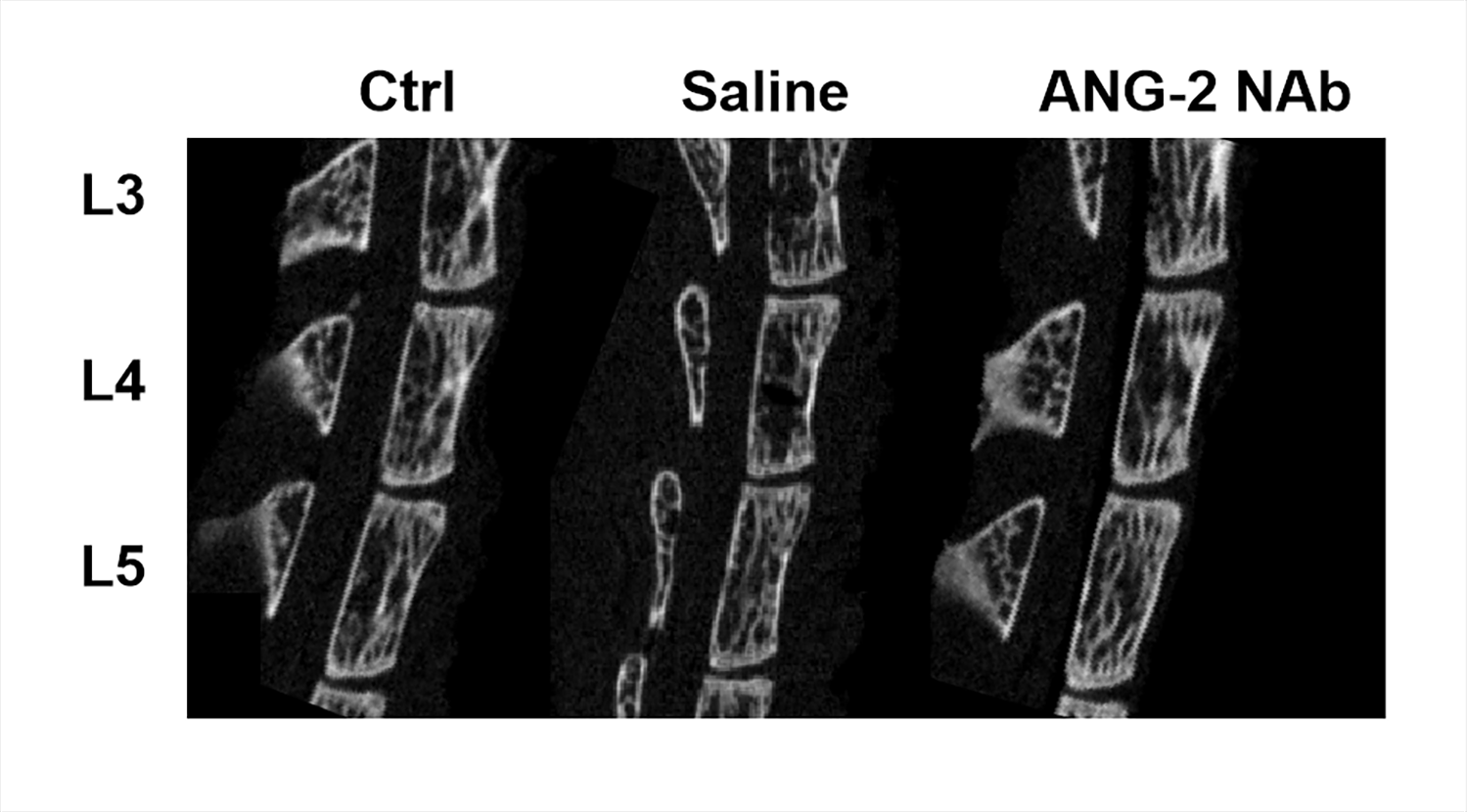

Supplement: Supplementary file 3 — Supplementary figure 3: The CT images of the lumbar spine tissues of the mice in the Ctrl group, the Saline group and the ANG‐2 NAb group. [file OS-15-2410-s001.tif]
